# Supplementary material for: Connecting Air Pollution Exposure to Socioeconomic Status: A Cross-Sectional Study on Environmental Injustice among Pregnant Women in Scania, Sweden
Source: Int J Environ Res Public Health. 2019 Dec 14;16(24):5116. doi: 10.3390/ijerph16245116 (PMC6949975; doi:10.3390/ijerph16245116)
Supplement: Supplementary file 1 [file ijerph-16-05116-s001.zip › ijerph-644225-supplementary.pdf]

Supplementary materials

Table S1. Descriptive information of MAPSS women's age frequencies among the total study population, Malmö and Lund.

	**Total Study Population (*n*= 48,777)**	**Malmö (n= 20,226)**	**Lund (n= 8,554)**
	% (*N*)	% (*N*)	% (*N*)
Age			
20 and younger	3.2% (1,584)	5.0% (1,005)	1.2% (102)
21- 25 years old	16.1% (7,833)	21.7% (4,386)	10.1% (864)
26- 30 years old	33.0% (16,083)	34.2% (6,916)	31.8% (2,719)
31- 35 years old	30.2% (14,723)	26.7% (5,406)	37.7% (3,226)
36- 40 years old	11.9% (5,822)	10.7% (2,167)	16.8% (1,435)
41 and older	1.8% (860)	1.7% (346)	2.4% (208)
Missing	3.8% (1,872)	-	-

- = No missing values to report.

Table S2. Annual average NO_X to NO_2 concentration (μg/m^3) conversions for pollutant summaries of main analysis by study area.

	**NO_X**	**NO_2**
Study area		
Total catchment area	17.8	16.9
Malmö	23.6	21.1
Lund	14.5	14.2

NO_X = Nitrogen oxides. NO_2 = Nitrogen dioxide. Swedish EPA's Clean Air objectives NO_2: 20 μg/m^3 [1]. Formula used: $NO_2 = NO_X (0.75 + (18 / (NO_X + 60)))$ [2].

Table S3. Annual average NO_X to NO_2 concentration (μg/m^3) conversions for pollutant summaries of Change over time sensitivity analysis by study setting.

	**1999–2005**		**2006- 2009**	
**Study Area**	**NO_X**	**NO_2**	**NO_X**	**NO_2**
Total catchment area	18.6	17.5	16.2	15.6
Malmö	25.5	22.4	20.5	18.9
Lund	15.3	14.8	12.2	12.2

NO_X = Nitrogen oxides. NO_2 = Nitrogen dioxide. Swedish EPA's Clean Air objectives NO_2: 20 μg/m^3 [1]. Formula used: $NO_2 = NO_X^{(0.75 + (18 / (NO_x + 60)))}$ [2].

Table S4. Descriptive table of a dichotomized version of *Birth country*: Swedish-born mothers and those born in Other countries for women in the MAPSS birth cohort living in Lund.

	**Frequency**
	% (*N*)
Maternal country of birth	
Sweden	80% (6,813)

Other country	20% (1,722)
Missing	0.20% (19)
Total	8,554

Table S5. Associations between levels of NO_X above Clean Air objective [a] and *Birth country* for women in the MAPSS birth cohort living in Lund.

	**OR (95% CI)**
	NO_X
Maternal country of birth	
Sweden	REF
Other countries	1.11 (0.94–1.31)

[a] Swedish EPA's Clean Air objectives NO_2: 20 μg/m³ [1]. OR = Odds ratio. CI = Confidence interval. NO_X = Nitrogen oxides. REF = Reference category.

Table S6. Sensitivity analysis on parity: Associations between exposure to levels of NO_X and $PM_{2.5}$ (μg/m³) above Clean Air objectives [a] and Household disposable income [b] for the 48,777 pregnant women of MAPSS living in Scania.

	**OR (95% CI)**			
	**NO_X**		**$PM_{2.5}$**	
	1 child	>1 child	1 child	>1 child
Household disposable income				
30,000–200,000	2.87 ** (2.65–3.10)	4.76 ** (4.38–5.17)	1.65 ** (1.44–1.89)	2.66 ** (2.30–3.07)
200,000–300,000	1.78 ** (1.64–1.93)	2.70 ** (2.50–2.92)	1.17 * (1.03–1.34)	1.52 ** (1.36–1.71)
300,000–400,000	REF	REF	REF	REF
>400,000	0.71 ** (0.64–0.77)	0.50 ** (0.46–0.56)	1.21 ** (1.07–1.38)	1.16 ** (1.05–1.29)

[a] Swedish EPA's Clean Air objectives NO_2: 20 μg/m³ and $PM_{2.5}$: 10 μg/m³ [1]. [b] Measured in Swedish kronor (SEK), annually. OR = Odds ratio. CI = Confidence interval. NO_X = Nitrogen oxides. $PM_{2.5}$ = Particulate matter with diameter ≤2.5 μm. REF = Reference category. * $p \leq 0.05$. ** $p \leq 0.01$.

Table S7. Sensitivity analysis on parity: Associations between exposure to levels of NO_X and $PM_{2.5}$ (μg/m³) above Clean Air objectives [a] and Household disposable income [b] for the 20,226 pregnant women of MAPSS living in Malmö.

	**OR (95% CI)**			
	**NO_X**		**$PM_{2.5}$**	
	1 child	>1 child	1 child	>1 child
Household disposable income				
30,000–200,000	1.57 ** (1.38–1.78)	2.28 ** (2.01–2.59)	0.99 (0.72–1.37)	1.56 ** (1.16–2.10)
200,000–300,000	1.65 ** (1.43–1.90)	2.15 ** (1.89–2.44)	0.98 (0.69–1.40)	1.19 (0.90–1.58)
300,000–400,000	REF	REF	REF	REF
>400,000	0.63 ** (0.54–0.73)	0.44 ** (0.38–0.51)	1.16 (0.78–1.72)	1.03 (0.74–1.44)

[a] Swedish EPA's Clean Air objectives NO_2: 20 μg/m^3 and $PM_{2.5}$: 10 μg/m^3 [1]. [b] Measured in Swedish kronor (SEK), annually. OR = Odds ratio. CI = Confidence interval. NO_X = Nitrogen oxides. $PM_{2.5}$ = Particulate matter with diameter ≤2.5 μm. REF = Reference category. * $p \leq 0.05$. ** $p \leq 0.01$.

Table S8. Sensitivity analysis on parity: Associations between exposure to levels of NO_X and $PM_{2.5}$ (μg/m^3) above Clean Air objectives [a] and Household disposable income [b] for the 8,554 pregnant women of MAPSS living in Lund.

	**OR (95% CI)**			
	NO_X		$PM_{2.5}$	
	1 child	>1 child	1 child	>1 child
Household disposable income				
30,000- 200,000	1.71** (1.32–2.21)	2.14** (1.52–3.02)	0.83 (0.60–1.15)	1.92** (1.29–2.86)
200,000- 300,000	1.54** (1.20–1.97)	1.99** (1.50–2.63)	1.02 (0.74–1.40)	1.29 (0.97–1.71)
300,000- 400,000	REF	REF	REF	REF
> 400,000	0.74* (0.56–0.97)	0.97 (0.72–1.30)	1.34 (0.99–1.83)	1.30* (1.03–1.63)

[a] Swedish EPA's Clean Air objectives NO_2: 20 μg/m^3 and $PM_{2.5}$: 10 μg/m^3 [1]. [b] Measured in Swedish kronor (SEK), annually. OR = Odds ratio. CI = Confidence interval. NO_X = Nitrogen oxides. $PM_{2.5}$ = Particulate matter with diameter ≤2.5 μm. REF = Reference category. * $p \leq 0.05$. ** $p \leq 0.01$.

Table S9. Pearson R correlations for all indices of socioeconomic status considered in the analysis.

	**Education level**	**Household disposable income [a]**	**Birth country**
Scania			
Education level	-	0.360 **	0.245 **
Household disposable income [a]	0.360 **	-	0.363 **
Birth country	0.245 **	0.363**	-
Malmö			
Education level	-	0.346 **	0.268 **
Household disposable income [a]	0.346 **	-	0.341 **
Birth country	0.268 **	0.341 **	-
Lund			
Education level	-	0.299**	0.169 **
Household disposable income [a]	0.299 **	-	0.246 **
Birth country	0.169 **	0.246**	-

[a] Measured in Swedish kronor (SEK), annually. ** Correlation significant at the 0.01 level (2-tailed).

References

1. EPA, S. Specifications for Clean Air.; Available online: http://www.swedishepa.se/Environmental-objectives-and-cooperation/Swedens-environmental-objectives/The-national-environmental-objectives/Clean-Air/Specifications-for-Clean-Air/. (assessed on: 16 April 2019)
2. Gustafsson, S., *Uppbyggnad Och Validering Av Emissionsdatabas Avseende Luftföroreningar för Skåne Med Basår 2001*. Institutionen för Naturgeografi och Ekosystemanalys: Lund, Sweden, 2007.
